# Enhancing Transitions From Rehabilitation Patient to Wellness Participant for People With Disabilities: An Opportunity for Hospital Community Benefit

**DOI:** 10.3389/fpubh.2020.00105

**Published:** 2020-04-08

**Authors:** Nathan W. Carroll, Allyson G. Hall, Sue Feldman, Mohanraj Thirumalai, Jamie Tinker Wade, James H. Rimmer

**Affiliations:** ^1^Department of Health Services Administration, University of Alabama at Birmingham, Birmingham, AL, United States; ^2^School of Health Professions, University of Alabama at Birmingham, Birmingham, AL, United States; ^3^Spain Rehabilitation Center, University of Alabama at Birmingham Hospital, Birmingham, AL, United States

**Keywords:** disability, community benefit, wellness, transitions in care, hospital, quality of life

## Abstract

Pressure is increasing on not-for-profit hospitals to demonstrate that they provide sufficient benefit to the community to justify their tax-exempt status. Many industry observers have suggested that this community benefit should address unmet medical needs within the community, deficits in the social determinants of health, or health disparities within communities. We argue that one area of clear unmet need is assistance in helping bridge the transition that people with disabilities (PWD) must make from rehabilitation patient to wellness participant. Programs to bridge this transition are necessary because many PWD struggle to identify strategies to maintain and maximize their own well-being after discharge from the healthcare system. As a result, PWD have worse health outcomes than non-disabled individuals. To address these needs, we propose hospitals take a leading role in establishing new, community-based efforts to provide PWD with benefits that will support their effort to self-manage health. Hospitals are well-suited to lead the creation of these programs because of the important role they play in providing services to PWD and because of their ability to bring together multiple stakeholders required to make supportive programs sustainable.

## Introduction

Regulators and other industry observers have recently suggested that not-for-profit hospitals should enhance their provision of community benefit (1). In some examples, these suggestions mean hospitals should be providing more charity care and/or outreach services (2). In other cases, calls for increased community benefit provision suggest hospitals do more to address the social determinants of health that can be barriers to improving health (3). Further still, requests for increased community benefit provision suggest hospitals should take action to address health disparities (4). Often, populations targeted as beneficiaries of “community benefit” programs have substantial resource constraints and limited access to the social determinants of health.

There is also another population whose health needs are being unmet, people with disabilities (PWD) (5). An estimated 30 million people in the U.S. have a mobility disability (6) accruing over $53 billion in direct medical costs annually (7). This population has higher rates of hospitalization, often for preventable conditions (8). For instance, patients with spinal cord injuries are hospitalized 2.6 times more often that similar individuals without disabilities, and a significant percentage of these hospitalizations are for preventable conditions like pressure ulcers, urinary tract infections or pneumonia (9). This largely underserved population has characteristics matching many of the motivations for community benefit provision. They often have medical needs that most physicians are unaware of how to treat (10–12), face social and environmental barriers to maximizing their health status and quality of life (13–15), and experience health outcomes far worse than individuals who do not have a disability (16–19). One of the primary factors contributing to these problems is that few local provider systems offer PWD a smooth transition from acute care or inpatient rehabilitation to community-based programs that empower them to control their own well-being. For hospitals that treat a significant number of PWD and have adequate resources, creating programs to support PWDs' efforts to self-manage their health is one rarely considered form of community benefit with the potential to make a significant impact.

Hospitals have unique capabilities to address many of these unmet needs and, through the provision of specific community benefits, can lead the way in creating comprehensive systems that provide care and supportive services enabling PWD to reduce rehospitalizations and emergency room care while improving their quality of life. In this paper, we argue that hospitals are well-positioned to convene groups of stakeholder organizations including rehabilitation centers, disability advocacy groups, and community resources. We suggest that hospitals lead coalitions of these stakeholder groups in addressing the needs of PWD within a hospital's local community. Further, we illustrate these points by describing the experience of one health system engaged in a community benefit program to improve PWD's transition from rehabilitation patient to self-managed wellness.

## Barriers in PWDs Transition From Rehabilitation Patient to Wellness Participant

One enormous challenge many PWD face after acquiring a disability or receiving medical care for a new secondary health condition (e.g., pressure ulcer and urinary tract infection) is transitioning back into the community and self-managing their health. Many individuals never make the transition from “patient” to “participant” (20, 21). They are anchored to a healthcare system focused on disease management, while their ability to self-manage health through wellness activities is usually non-existent.

There are internal and external barriers that inhibit PWD from engaging in self-management. These include a lack of information about how to manage health while living with a disability, a lack of access to community-based healthcare providers who understand mobility disabilities, financial challenges finding support for daily activities, transportation barriers, and a lack of social support from friends and family (5, 12, 13, 15, 22–25).

PWD often experience barriers to exercise and wellness beyond those experienced by the general population, including cost of fitness facility membership, access to public transportation, lack of information on accessible facilities and programs, lack of accessible exercise equipment, physical layouts challenging to people using mobility devices, and the perception that fitness facilities are unfriendly environments for PWD (26–31).

There is more that hospitals can do to ensure PWD experience a smooth transition from hospital care to community care. An ideal time to capture the attention and awareness of individuals who have acquired a new mobility disability (e.g., stroke, head injury, spinal cord injury, and limb loss), new diagnosis (e.g., multiple sclerosis and Parkinson's), or are receiving medical care for a new or recurring secondary health condition (e.g., joint pain, fatigue, edema, type 2 diabetes, reduced balance, pressure ulcer, urinary tract infection, and depression), is when they are receiving hospital care. This is often a time when they and/or their caregiver are aware of the need to improve their health after they return home from the hospital or healthcare facility (32, 33). Patients develop a trusting relationship with healthcare providers and may look to these individuals for guidance on how to maintain their health while outside the hospital.

Aside from interacting with patients during critical points in their recovery, hospitals have another unique characteristic that could improve the reach of wellness programs serving PWD. Relative to some of the community-based, voluntary organizations currently providing services, hospitals have a high degree of organization, administration, and sustainability. These characteristics could allow hospitals to help establish new systems that integrate the health services and community health portions of the care continuum; systems that would be difficult to achieve in less-structured collaborations between organizations currently serving PWD. For instance, hospitals' capacity for administration will be critical in establishing new approaches to data-sharing between providers that will be necessary to support a smoother care continuum for PWD.

Hospitals seeking to support the promotion of wellness among PWD as they reenter the community will find that there are a growing number of national organizations that could help sustain this important effort. For instance, the National Center on Health, Physical Activity, and Disability (NCHPAD) has been funded by the Centers for Disease Control and Prevention (CDC) for the past 20 years and has been active in developing resources to help communities become more inclusive places for PWD to pursue healthy lifestyles. These efforts include the development of the MENTOR program (Mindfulness, Exercise, and Nutrition to Optimize Recovery). MENTOR is a health coaching platform that is targeting PWD who have had a recent interaction with the healthcare system.

NCHPAD has also been active in supporting the efforts of stakeholder groups within 16 communities to coordinate efforts to improve the lives of PWD. The groups participating in these inclusive health coalitions (IHCs) are primarily volunteer and community-service-focused organizations. The IHC effort has yielded notable improvements in the locations where IHCs exist, and the MENTOR effort has the potential to bring wellness benefits to PWD in communities across the U.S. However, partnerships with hospitals could dramatically increase the ability of these, and other existing programs, to serve PWD. Hospital partnerships could help community-based organizations connect with recently-diagnosed PWD earlier in their treatment process to create a smoother continuum between rehabilitation/healthcare and community-based wellness. Hospitals could also provide community-based organizations with the organizational support and funding required to pursue more ambitious strategies for providing benefits and organizing information technology infrastructure.

## A Community Benefit Program Tailored to PWD

While the need for additional support to PWD is real, and the potential for hospitals to meet this need is significant, there are relatively few examples of hospitals engaging in efforts to meet the post-discharge needs of PWD. However, for the past several years, one of the authors has lead an effort by a large academic health system in the southeastern United States, to meet the post-discharge needs of PWD. This health system has been in partnership with a not-for-profit organization (NFPO) dedicated to improving the lives of people with physical disabilities through physical activity and wellness. Working together, these two organizations have pilot tested several methods to transition PWD from skilled therapy to a community wellness program designed to improve patients' ability to self-manage. We offer detail about these efforts and insights gained from different approaches tested.

### Benefits Provided

Initially, participants received skilled outpatient therapy services provided by the health system. Interventions included gait training, functional transfers, community outings, driving rehabilitation, and aquatic therapy, which were all delivered by a multidisciplinary team of occupational, physical, recreational, and speech therapists. All community activities and therapy interventions were provided with the goal of improving individuals' ability to function in the community setting and participate in a lifestyle of wellness. Those interventions were not often covered by participants' insurance, either because the therapy visits exceeded annual limits or because the category of therapy (e.g., in-car driving therapy) is not covered by insurance. The ability to engage in non-covered therapy made a valuable contribution to furthering participants' wellness. Driving therapy increased participants' independence. Recreational therapy (defined as therapy with the goal of helping individuals with functional limitations learn to engage in activities they enjoy) was helpful in improving participants' quality of life and served as a vehicle to pursue clinical goals like improving memory and cognition.

The second part of the pilot program offered participants a structured transition from pursuing outpatient therapy under the supervision of a skilled therapist (e.g., occupational or physical therapist) to pursuing therapy goals outside of skilled therapy sessions, in a fitness facility. The exact form this transition took changed over time, as therapy staff tested new transitions and adapted their approach. Initially, health system therapists provided PWD with written materials and education on the programs available at the NFPO. This approach appeared to be ineffective, with little follow through from PWD because they did not have a clear idea what services were available and which were appropriate for them given their unique mobility limitations and therapy goals. Health system therapists worked to improve the transition process by partnering with occupational therapy graduate students who took participating PWD on visits to the NFPO to more formally introduce them to resources available. Again, the transition was not as successful as anticipated. PWD participating in the pilot were still not consistently utilizing the NFPO recreational facilities.

Mostly recently, the health system and NFPO began experimenting with “warm transitions” from health system therapy to the NFPO. This transition model involves outpatient therapists from the health system meeting with fitness facility staff and pilot participants. The goals are to introduce pilot participants to fitness facility staff, and to identify fitness center activities that would help further goals set by the participants. Early results suggest that these “warm transitions” are successful ways to increase participation in activities that support the wellness of PWD. However, these structured handoffs did require a significant time commitment from outpatient therapy staff that was unreimbursed and that goes beyond the scope of services typically provided.

Program staff have identified a number of additional benefits that would have helped enable participants to pursue wellness goals. These included additional support for participants, family, and caregivers. Additional support for participants could include personalized help navigating care coordination issues or additional emotional support during the transition from skilled therapy to independent health management. In the future, the health system hopes to pair participants with peer “ambassadors” who have diagnoses similar to the participants they are assisting, and who have successfully navigated challenges of care coordination and self-management. Health system staff have also identified the need for benefits to support the family and caregivers of participating PWD, including respite care opportunities. These are especially needed for caregivers assisting in the care of a participant while also caring for children or aging parents. Activities like maintaining a network of peer ambassadors or arranging and funding child care all require the administrative capabilities hospitals possess, and could be valuable ways to provide community benefit that meets the needs of PWD.

### Factors That Facilitate Program Success

Efforts to create a smoother care continuum were led by members of the health system's outpatient therapy department. Three factors have played a critical role in the success of these efforts: choosing the right participants, the availability of resources within the community, and the commitment of both the health system and its community-based partner (NFPO) to the program. Program staff note that participants who are emotionally ready to take responsibility for the management of their health, and intrinsically motivated to participate seem to be more successful. This was an important realization since, for some PWD, it can take a few months to a few years after acquiring a mobility disability before being prepared to engage in a program like this one. In addition, initial program efforts focused on creating transitions for participants with few comorbidities requiring medical management, though future interventions may include more medically complex participants.

Another key to the success of the health systems' initial efforts has been offering access to a wide variety of community-based opportunities. For program participants, the NFPO offered several daily classes (e.g., fitness, yoga, balance, and Thai Chi) fully adapted to the needs of PWD. These resources allowed participants to choose activities that would foster progress toward their goals. Finally, both the NFPO and the health system committed to making the program work. This commitment is critical since the “warm transitions” that were most successful required changes to existing workflows and staff roles for individuals at both organizations.

### Barriers Identified

Through the pilot, the health system identified several barriers to program success. Other hospitals looking to provide similar services are likely to face similar barriers. One of the primary barriers will be identifying funding for program services. The pilot population had funding from a unique source not available to most patients, but obtaining funding is likely to be a challenge. Many of the skilled therapy services are not covered by most insurance plans either because of limits on the annual number of therapy visits covered or because some skilled therapies are not covered at all. Other health systems pursuing this kind of community benefit program will have to identify the extent of their financial commitment to the program and may look to supplement the funding they provide with other sources of funding within the community.

Another barrier identified was communicating the health system's goals for the program to partnering NFPOs. Even though leaders at the health system and the NFPO agreed on a shared vision, communicating this vision to front-line staff was a challenge. Communication between health system and NFPO staff was also a problem. Initially, the two groups did not always understand each other's rolls. This problem was compounded by the lack of effective mechanisms to communicate information about participants' care plans, progress, and barriers. Ideally, the program would have used technology to facilitate sharing information about the participants' experience in the program. In addition, the health system and NFPO are considering ways to facilitate an improved understanding of the roll each group's staff members play. Other health systems could foster this understanding through unique forms of community benefit like providing health system therapists paid time to shadow staff at partnering community organizations.

### Adapting the Pilot to Other Health System Settings

Several aspects of this program may be unique to the health system that began implementing it. Most important, the health system had the support of an NFPO with unusually deep experience working with PWD. As a result, health systems replicating this kind of community benefit program may need to consider ways to help their community partners (for instance, community recreation centers, or YMCAs), understand how to serve the needs of PWD. Resources to support this effort are available through the NCHPAD. For example, NCHPAD and the American College of Sports Medicine have developed a certification as an Inclusive Fitness Trainer. Similarly, NCHPAD is currently implementing an online health coaching program aimed at the needs of PWD (MENTOR–Mindfulness, Exercise, Nutrition To Optimize Recovery). Health systems will also need to address participant intake. Large numbers of participants are likely to complicate information transfer and participant selection. The health system described found that even with a limited participant population, a comprehensive intake process was required. The intake process should document participants' level of function, support systems, expectations of the program, interests, and activities in which they hope to participate. In addition, the intake process should include representation from both the health system and community partner. Finally, the intake process should set reasonable participant and family expectations and should document the roles and responsibilities of each team member, the participant and family members.

**Figure 1:**
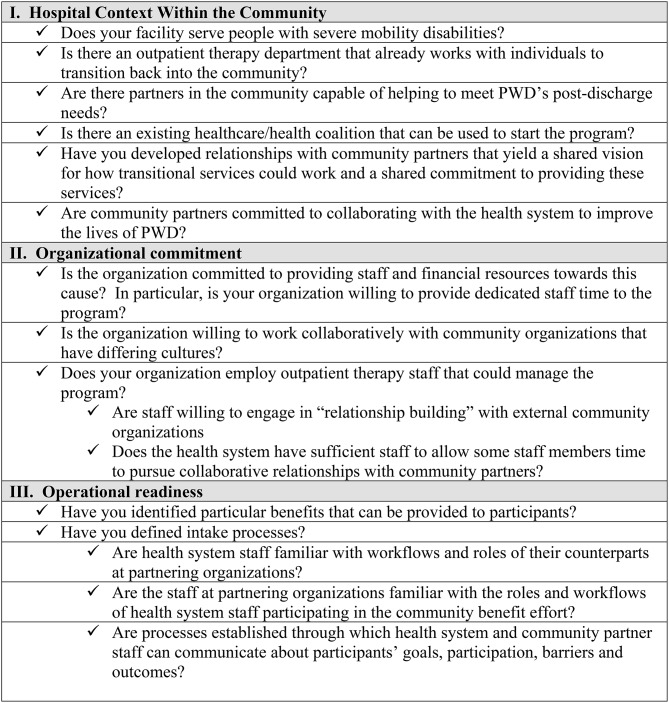
Checklist for Hospital Preparedness. The following questions will help hospitals assess their readiness to begin offering a program to promote wellness among PWD within the community. The checklist identifies important hospital and community factors as well as operational questions that will need to be addressed. Hospitals should pay special attention to the “organizational commitment” questions, since this sort of program cannot succeed without identifying the program as a priority and providing staff and financial resources to support it.

In Figure 1, we summarize the pilot program's lessons about the hospital characteristics required to successfully implement a community benefit program to promote wellness among PWD living in the community.

## Discussion

Hospitals are increasingly pressured to provide community benefits and to show that their efforts are making an impact on their communities. We argue that hospitals can have a significant impact on the lives of PWD by working to assist these individuals in making the transition from rehabilitation patient to wellness participant. This form of community benefit will require hospitals to provide initial financial funding and organizational support, acting as catalysts to bring together community stakeholders, many of whom may already be working to improve the lives of PWD. This role plays to competencies that hospitals possess, like extensive administrative capabilities and the ability to coordinate efforts between multiple stakeholders including healthcare providers, advocacy groups, and patients themselves.

One way for hospitals to consider reaching PWDs is to create or join an Inclusive Health Coalition (IHC) that galvanizes a community around key issues of need in promoting community health inclusion. Hospitals should evaluate their community for these types of potential partnerships. IHCs offer an existing organizational structure that can support the provision of wellness services. By providing these services and engaging healthcare delivery organizations in creating and disseminating these programs, IHCs can make progress in creating a unique continuum of care that meets the needs of individuals who have recently accessed the healthcare system. We propose that individual IHCs expand their missions to include a specific focus on patient transitions from healthcare to wellness, helping patients progress from medically managed care in which they receive services from healthcare providers, to full-fledged participation in long-term wellness.

## Author Contributions

All authors contributed to the conceptualization of the article. NC drafted the manuscript and AH, SF, MT, JW, and JR provided critical feedback and made substantive changes which improved the manuscript.

### Conflict of Interest

The authors declare that the research was conducted in the absence of any commercial or financial relationships that could be construed as a potential conflict of interest.
